# Dietetic interns’ perceptions and use of evidence-based practice: an exploratory study

**DOI:** 10.5195/jmla.2018.308

**Published:** 2018-01-02

**Authors:** Rachel J. Hinrichs

## Abstract

**Objective:**

This study explored dietetic interns’ perceptions and knowledge of evidence-based practice (EBP), their use and observation of EBP principles during their clinical rotations, and their intentions to use EBP in their careers.

**Methods:**

A mixed methods design combining a survey and focus group was employed. Dietetic interns (n=16) from a large Midwestern university were recruited in person and via email to participate in the survey, focus group, or both. Perceptions and experiences of EBP were analyzed through the focus group (qualitative), and EBP knowledge and clinical practice behaviors were analyzed through the survey (quantitative). The focus group discussion was recorded, transcribed, and analyzed using thematic analysis.

**Results:**

Four major themes emerged from the focus group data: (1) observations of EBP in clinical practice, (2) use of EBP during clinical rotations, (3) barriers to EBP, and (4) perceived use of EBP as future registered dietitians. Interns considered EBP important for their profession and future careers. They struggled, however, with the discrepancies between current research and practice, and highlighted differences that they observed and barriers that they experienced across different clinical settings.

**Conclusions:**

This exploratory study is the first to examine dietetic interns’ perceptions of and experiences with EBP in the clinical setting. Future research is needed to identify how dietetics educators, librarians, and preceptors can address the barriers that interns perceive in applying EBP in their internships.

## INTRODUCTION

Nutrition and dietetics is one of many health care fields to recognize the importance of evidence-based practice (EBP) in supporting clinical decision making [1]. The Academy of Nutrition and Dietetics defines evidence-based dietetics practice as “the process of asking questions, systematically finding research evidence, and assessing its validity, applicability and importance to food and nutrition practice decisions” in light of the client’s or patient’s values [2]. Described as a core competency of dietetics, EBP can assist registered dietitians (RDs) in bridging the gap between research and practice, improving quality of care and performance of health professionals, and obtaining reimbursement for services [1, 3].

To support RDs’ use of EBP, many resources have been developed, including EBP guidelines [4], a library of pre-appraised nutrition evidence [5], and continuing education classes [6]. Nevertheless, while RDs have favorable perceptions of EBP, implementation in clinical practice has been slow and inconsistent [7–10]. Surveys report that RDs use EBP resources less than once per month and rely on colleagues, websites, and continuing education resources for information [10, 11]. Barriers to EBP include lack of time, training, and mentors [10].

There appears to be a need to incorporate EBP into dietetics education. To become a RD, a student must earn a bachelor’s degree, take particular courses specified by the Academy of Nutrition and Dietetics, complete a supervised practice dietetic internship, and pass a national registration exam [12]. The 2017 Accreditation Standards for Dietetic Internship Programs state that the curriculum must cover EBP principles [13]. This includes the application of clinical guidelines and scientific literature to practice and critical appraisal of literature [13]. Students need to have strong research, literature searching, and critical appraisal skills in order to use and ultimately build on the evidence base in their fields.

While several articles addressed teaching research skills to nutrition and dietetics students, few focused specifically on teaching EBP [14–16]. Three case studies of librarian-led instruction with undergraduate students on EBP and information literacy suggested that library instruction increased student use of library resources and skills in literature searching [17–19]. No studies, however, addressed librarian involvement with dietetic interns. Further, no studies addressed dietetic interns’ perceptions of EBP and their experiences using EBP during their clinical rotations.

The purpose of this pilot study was to explore dietetic interns’ perceptions and knowledge of EBP, their use and observation of EBP principles during their clinical rotations, and their intentions to use EBP in their careers. The goal was to inform EBP instruction for future dietetic interns, including identifying where librarian involvement is most needed. A deeper understanding of the barriers and challenges to EBP that dietetic interns face during their internships will enable instructors and librarians to design more relevant and practical curricula.

## METHODS

A mixed methods design combining a survey and focus group was employed to explore dietetic interns’ experiences with and perceptions of EBP. Mixed methods, particularly those combining qualitative and quantitative methods, allow researchers to study phenomena that are complex and multifaceted in a more complete and robust way [20]. The outcomes assessed were perceptions and experiences of EBP through a focus group (qualitative) and knowledge and clinical practice behaviors through a survey (quantitative).

Dietetic interns (n=16) from a large Midwestern university were recruited in person by a health sciences librarian and via email to participate in an anonymous survey, a focus group, or both. The participants were told that the purpose of the study was to understand their experiences with EBP in order to improve the curriculum and library sessions for the next year. As an incentive, the interns were offered extra credit points for completing the survey and a $10 Amazon gift card for completing the focus group. This study received institutional review board (IRB) approval prior to beginning data collection.

### Survey

The survey instrument was originally developed and used by Vogt and colleagues to measure RDs’ EBP knowledge and clinical practice behavior before and after an EBP educational session [21]. The first section of the survey on EBP knowledge consisted of free-text (2-point), multiple choice (1-point), and true/false (0.5-point) questions with a maximum knowledge score of 12. An independent-samples *t*-test was used to compare the interns’ scores in the present study with the registered dietitians’ scores in Vogt and colleagues’ study [21]. Statistical significance was defined as a *p*-value <0.05.

The second section of the survey on clinical practice behavior measured how often certain EBP behaviors were performed. For the present study, the scoring was modified from the original instrument to use a 5-point Likert scale (5=2 or more times per week; 4=once per week; 3=one to 3 times per month; 2=less than once per month; 1=never) with a maximum clinical practice score of 45. High clinical practice scores indicated a greater implementation of EBP clinical behaviors. The survey was administered anonymously using Qualtrics. The link to the study was distributed to the interns in April 2017, 2 months prior to the end of their internship, and was left open for 2 weeks.

### Focus group

The author facilitated the focus group a week after the survey was distributed in April 2017. Focus group participants were volunteers. Questions were developed beforehand to guide the discussion (supplemental appendix), and follow-up questions were asked during the focus group to delve deeper into the participants’ responses. The session was recorded, transcribed, and analyzed by the author using thematic analysis, which helped to avoid bias by ensuring that the themes were grounded in the participants’ responses to the questions [22]. This process involved first coding and re-coding the transcript with notes relevant to the research goal and objectives. Codes were then grouped to develop themes, while referring back to the original transcript to capture the original meaning and context.

## RESULTS

### Survey

The survey was completed by 14 out of 16 interns, which is a response rate of 88%.

#### Evidence-based practice (EBP) knowledge

Dietetic interns scored a mean of 7.75 out of a possible score of 12 (65%) on the EBP knowledge portion of the survey. Compared to the RDs at baseline in Vogt and colleagues’ study [21], dietetic interns were found to have similar EBP knowledge item scores (Table 1). Both groups had lowest scores on the statistics and patient or problem, intervention, comparison, outcomes (PICO) definition questions. The RDs received significantly higher scores than dietetic interns for total knowledge (*t*(34)=2.2, *p*<0.05) and composition of a PICO question (*t*(34)=3.9, *p*<0.05).

**Table 1:** Evidence-based practice (EBP) knowledge item and total scores (mean ± standard deviation) of dietetic interns and registered dietitians

Knowledge score	Maximum score per item	Dietetic interns, n=14	Registered dietitian: intervention group baseline, n=22*
Interpret statistical results	0.5	0.04 ± 0.13	0.07 ± 0.18
Determine statistical results	0.5	0.14 ± 0.23	0.25 ± 0.26
Patient or problem, intervention, comparison, outcomes (PICO) refers to	0.5	0.17 ± 0.25	0.20 ± 0.25
Boolean terms reduce citations	0.5	0.32 ± 0.25	0.32 ± 0.25
First-step EBP model	0.5	0.36 ± 0.23	0.41 ± 0.20
Medical Subject Headings (MeSH) terms reduce citations	0.5	0.43 ± 0.18	0.45 ± 0.15
Determine sound research	1	0.64 ± 0.50	0.77 ± 0.43
Most appropriate study type	1	0.79 ± 0.43	0.95 ± 0.21
Highest study in hierarchy	1	0.79 ± 0.43	0.82 ± 0.39
Compose PICO	2	1.07 ± 0.65	1.82 ± 0.50
Characteristics of sound randomized controlled trial	2	1.39 ± 0.56	1.45 ± 0.43
Valid EBP resources	2	1.61 ± 0.21	1.45 ± 0.43
Total knowledge score	12	7.75 ± 1.34	9.02 ± 1.85

* Data from the study by Vogt and colleagues [21].

#### EBP clinical practice behaviors

Dietetic interns reported that they often performed clinical practice behaviors associated with EBP (Table 2). The behaviors performed most often, on average one or more times per week, were accessing nutrition information through professional association or government websites and accessing medical evidence in general. Journal articles were accessed more often than Cochrane systematic reviews or pre-appraised resources. Critical appraisal, writing a searchable PICO question, and accessing the Cochrane Library were performed the least, on average less than once per month.

**Table 2:** Clinical practice behavior item and total scores (mean ± standard deviation) of dietetic interns

Clinical practice behavior scale	Maximum score per item	Dietetic interns, n=14
Access nutrition websites (government or professional associations)	5	4.4 ± 0.8
Access medical evidence in general (journal articles, databases, clinical guidelines)	5	4.1 ± 1.1
Access original research articles	5	3.6 ± 1.0
Answer background questions using databases	5	3.4 ± 1.3
Use EBP to change practice	5	3.1 ± 1.5
Access pre-appraised evidence (Evidence Analysis Library, clinical guidelines)	5	3.0 ± 1.2
Critically appraise research	5	2.6 ± 1.0
Write a searchable question	5	2.6 ± 1.2
Access Cochrane Library	5	2.1 ± 1.0
Total clinical practice score	45	29 ± 7.2

### Focus group: summary of major themes

Seven interns (n=7, 44%) participated in the focus group. Because the survey was anonymous, it is unknown whether these interns also completed the survey.

#### Observations of EBP in clinical practice

Before they joined the internship program, most of the interns had heard the term “evidence-based practice” from their undergraduate classes and were familiar with the principles. Similar to the traditional definition, interns defined EBP as practice based on research and evidence.

I’d say [EBP is] practicing based on research. As opposed to your opinion or personal beliefs.

Despite their positive attitudes toward EBP, interns struggled with the discrepancies that they observed between current research and practice. While their classwork focused on the most up-to-date treatments, their experiences during their clinical rotations reflected the difficulties of changing practice to meet current research.

Everything in class is kind of the opposite [of what we do in rotations]. I guess it’s changing practice.

Sometimes I feel like there is a lag period between research and when it’s actually applied.

Interns reported that application of EBP principles varied across different clinical settings. Interns observed that RDs in critical care units, in particular the neonatal intensive care unit (NICU), consistently accessed new research and information, and discussed new studies among each other and with them.

I’ve seen preceptors, especially in NICU, are constantly I feel like looking up new research but I don’t think it’s super common. I know that those preceptors have piles on piles of evidence.

I noticed [NICU staff] looked up more stuff. And had more articles for me to read and study and do critical analysis.

When I was with the metabolic dietitian, I felt like there were a lot of rare genetic diseases that I had never heard of before I started this position. [The dietitians] are constantly having to research what those diseases are.

Because interns were placed in different hospitals, they discussed with each other the differences that they observed in practice between the hospitals, such as the practice of physically touching patients to do nutrition assessment.

It’s just interesting to see at different hospitals how different practice is, even between different facilities.

#### Use of EBP as interns during their clinical rotations

While interns searched the literature and analyzed studies as part of their classwork, their use of EBP during their clinical rotations varied, largely depending on their clinical preceptors. Some preceptors encouraged their interns to be a part of the process by searching for the latest research evidence or applying it to their patients.

I had a burn unit preceptor that said “What are they telling you now?” And she’s been doing it for a while. And she was asking me.

I looked them up [articles on a particular therapy], because she said she hadn’t seen anything in the literature yet. So I looked it up because I had free time.

Many of their preceptors, however, were not as eager or able to provide interns with the opportunity to apply EBP to their clinical rotations.

My preceptor had a conversation with me that she’s the RD and I’m the intern. And so I shouldn’t be questioning the way she does things.

I feel like we go through trying to please our one preceptor. And so we don’t want to bring in new information. We want to copy what they are doing.

It’s not really up to us. We can’t really apply [EBP].

As students, the interns felt that they should focus on observing and copying their preceptors. Even so, the opportunities to be a part of the process and assist their preceptors were positive experiences and actively engaged the interns in applying EBP principles to a real case.

#### Barriers to EBP

Interns identified several barriers to EBP including time, access to resources, interprofessional conflict, and resistance to change. Certain EBP steps such as accessing resources and critical appraisal were thought to be difficult to do on a regular basis because of the time required.

I think finding time to decipher which [studies] are good or not. The assignments we had in class where we had to figure out whether this worked or not took us a really long time. And so I can’t imagine [doing it during my job].

In some places, [articles] were harder to find. Because for some of my preceptors, it was hard for us to find computers.

Interns discussed their concerns about how to access journal articles upon leaving the university. Several of them mentioned observing clinicians using Google for answers to clinical questions.

Interprofessional conflict, in particular with physicians, was also discussed as a key barrier. Interns noted disagreements over nutrition education and therapies.

I’ve looked up stuff, certain diets that doctors want to recommend. And then the dietitian hears about it, and it’s not something they would do. So then you have to figure out, to tell them there is no research to support what you’re doing here.

Pre-op[eration] [nutrition therapy] is changing pretty quickly…which research supports, but a lot of places—most places—it hasn’t been implemented. Mostly, probably, physicians are why…They say, “This has been working for me forever why would I change?”

With the exception of RDs in critical care, some interns believed that many RDs were resistant to changing their practice as well.

I guess it’s like registered dietitians fall into a groove of things. I mean every patient is individualized, but what you do in this situation, just doesn’t seem to change.

Interns thought that in order for a change in practice to occur, RDs, physicians, and other clinicians would need to be in agreement in appreciating the benefits that the change would bring.

#### Perceived use of EBP as future registered dietitians

Despite the barriers discussed, interns believed that they would base their future clinical practice on research and evidence. They readily perceived the need for using evidence to make decisions, especially for emerging research areas.

I think [EBP] would affect how we interpret new research. Like emerging nutrition topics. That some people are really quick to say, “Oh look they just found this.” And you can’t just say that, you have to go look into it. Look at how they did the study.

While sometimes frustrated, interns appreciated being able to observe different methods to approaching EBP and clinical problems, on both individual and organizational levels.

We want to copy what [our preceptors] are doing…And then decide for ourselves later. If we liked how this preceptor did things, and how this person did things.

[Practice] will change depending on where you go. There are just different ways of doing things.

**Figure 1:**
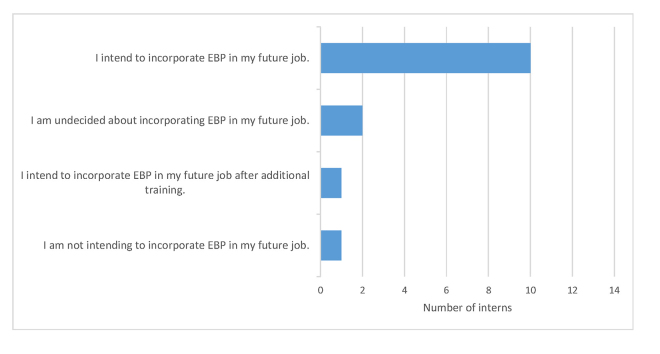
Dietetic interns’ intentions to use EBP in their future jobs (n=14)

These findings are also consistent with the survey results, where 72% (n=10) of the interns indicated “I intend to incorporate EBP in my future job” (Figure 1).

## DISCUSSION

In this mixed method study, dietetic interns discussed the significance of EBP in their clinical rotations and to their future careers. EBP and application of research were regarded as crucial for their profession and in provision of high-quality nutrition care for patients. In the last few months of their clinical rotations, interns had similar levels of EBP knowledge as RDs in clinical practice, except for composing a PICO question [21]. As indicated in both the survey and focus group, the most difficult part of the EBP process was critical appraisal, especially in regard to interpreting statistics. This is a common finding in EBP studies with students in other health fields [23–25].

The interns experienced and observed many barriers to and inconsistencies with EBP during their clinical rotations. The most common discussion topics throughout the focus group were the differences in practice between hospitals and hospital units. These differences were a source of interest and confusion, as interns considered how to reconcile the research they were reading in class with the reality they faced in the clinics.

Some of the interns did not feel like they had an opportunity to apply evidence during their rotations. The opportunity to use EBP in clinical practice seemed to be largely determined by the preceptors. While some preceptors encouraged interns to seek out new evidence on cases, others did not. This was similar to findings from studies on student nurses, physicians, and physical therapists [23, 24, 26]. Some clinicians appeared to promote EBP principles with their interns, while others focused more on clinical expertise and observation [24].

Assisting interns with EBP during their clinical rotations EBP may be a missed opportunity according to some nutrition educators. For example, Krantz and colleagues suggested that dietetic interns could act as resource for preceptors in learning about and applying the nutrition care process, a standardized method to apply nutrition evidence to practice [27]. In this study, the interns spoke positively about the opportunity to assist their preceptors with finding and evaluating new evidence. Interns may have more time and access to resources than their preceptors and so can help alleviate some of those barriers.

A deeper understanding of the barriers and challenges to EBP that dietetic interns face during their clinical internships can inform library instruction. While librarians cannot address all of the barriers that interns face, there are a number of actions that librarians can take to enrich and improve the interns’ experiences, as suggested by this study:

1. Partner with dietetic internship directors. Previous case studies have shown success in librarians collaborating with nutrition faculty who teach undergraduates [17–19]. Extending these partnerships to dietetic internship directors is a great opportunity for further support. Dietetic internship directors are required to demonstrate that their programs cover research and EBP. Librarians can help accomplish this by teaching literature searching, EBP principles, and information literacy concepts.
2. Focus on pre-appraised or summative resources, especially ones specific to their disciplines. Time was a frequently cited barrier to EBP. Interns voiced their concerns that they would not have enough time to search for articles and then appraise them while they are practicing. While librarians cannot grant more time, they can emphasize resources with pre-appraised evidence because these resources assist busy clinicians with finding high-quality evidence quickly. By the time they graduate, interns should be as familiar with the Cochrane Library, Turning Research Into Practice (TRIP), and Database of Abstracts of Reviews of Effects (DARE) databases as they are with PubMed. Furthermore, interns should be aware of pre-appraised resources specific to their disciplines. RDs have a unique database of pre-appraised research available to them through the Academy of Nutrition and Dietetics called the Evidence Analysis Library (EAL) [5], which provides evidence-based guidelines, bibliographies, and grades of evidence for various studies. Surprisingly, the interns in this study did not know of its existence. Highlighting this database could be helpful in encouraging interns to look for higher levels of evidence directly relevant to their field.
3. Address access to resources after graduation. The interns in this study were concerned about access to resources after they left the university and, in some cases, access to resources while in the clinics. This may be a good opportunity to discuss open access, publically available databases such as PubMed Central, and the role of the National Network of Libraries of Medicine and public libraries in providing access to biomedical information. The interns also suggested that an advanced searching session using Google and Google Scholar would help them identify freely available sources more efficiently. To address access to resources while in the clinics, interns can be provided with tablets with mobile applications or their awareness of mobile resources can be increased to help alleviate this barrier.
4. Provide resources to assist with critical appraisal. Much of the newest research in nutrition is not going to be covered in guidelines or pre-appraised resources. Understanding the fundamentals of critical appraisal is necessary for RDs to interpret emerging research that is important to their field or that their clients bring to them. Nutrition and dietetics educators will likely cover critical appraisal in their curricula, but librarians can make sure that they are aware of critical appraisal tools, checklists, and tutorials that the interns could use during and after their internships.

There were several limitations to this study. Convenience sampling was done using volunteers. This approach might introduced selection bias, because students who volunteered to participate might have had different perceptions of and experiences with EBP. No action was taken to obtain data on why other students did not volunteer for the focus group. A librarian conducted the focus group, which could have led to the participants commenting on resources, research, and literature searching differently than they would have otherwise. The participants were assured that their comments would have no effect on grades or completion of their internships. Finally, the sample size was small and not necessarily generalizable to all dietetic interns. The experiences reflected in this study could be unique compared to other dietetic internships.

This methodology of using a focus group and survey to understand students’ perceptions of and experiences with EBP in a clinical setting could interest health sciences librarians who support students in clinical-based internships or supervised practice. Clinical rotations expose students to the nuances and difficulties of translating research into practice. Librarians can help prepare future clinicians by addressing the barriers that they will face in applying evidence to practice.

Future research could look into whether instruction on pre-appraised resources versus on critical appraisal is more effective in easing the time and access barriers that busy clinicians encounter. Understanding student experiences on a deeper level can help librarians identify areas where their instruction and support is most needed and is influential beyond the classroom.

## Supplemental File

Appendix Focus group questions
