# Supplementary material for: Dietetic interns’ perceptions and use of evidence-based practice: an exploratory study
Source: J Med Libr Assoc. 2018 Jan 2;106(1):65–73. doi: 10.5195/jmla.2018.308 (PMC5764595; doi:10.5195/jmla.2018.308)
Supplement: Appendix [file 106-65-s001.pdf]

JMLA Journal of the Medical Library Association

SC

Supplemental content to
J Med Libr Assoc. Jan;106(1):dx.doi.org/10.5195/jmla.2018.308
www.jmla.mlanet.org
© Hinrichs 2018

Dietetic interns' perceptions and use of evidence-based practice: an exploratory study

Rachel J. Hinrichs, MS, MSLS, AHIP

APPENDIX

Focus group questions

1. [EBP topic introduction] What is the first thing that comes to mind when you hear the phrase "evidence-based practice" (EBP)?
   a. What did you know about EBP before starting the internship?
   b. Did you have any research experience prior to the internship?

2. [EBP 5As] Take a look at these steps in the EBP process. [Show a chart of the EBP process—Ask, Acquire, Appraise, Apply, Assess—with brief descriptions.]
   a. Which of these steps are you most comfortable with?
   b. Which of these steps are you the least comfortable with?

3. [Rotations] What types of questions came up during your rotations for which you needed to seek information to get an answer?
   a. What resources did you use to get answers, if any? What resources did you have access to?
   b. What resources did the preceptors provide or recommend, if any?
   c. What factors play a role in your decision to use various sources?
   d. How did you use the information you found? How do you determine its quality?

4. [Barriers] What barriers or challenges did you encounter as part of your internship?

5. [Library support] What can the library do to better prepare you for your rotations and research proposal?
